# Assessing the Preservation of Lumbar Lordotic Curvature in Everyday Sitting Conditions Assessed with an Inertial Measurement System

**DOI:** 10.3390/jcm13092728

**Published:** 2024-05-06

**Authors:** Ju Chan Kim, Jeong-Gil Kim, Beom Suk Kim, Cheol Ki Kim, Minseok Choi, Joonnyong Lee, Sun Gun Chung

**Affiliations:** 1 Department of Rehabilitation Medicine, National Traffic Injury Rehabilitation Hospital, Yangpyeong 12564, Republic of Korea; davidjckim91@gmail.com; 2 Department of Rehabilitation Medicine, Armed Forces Yangju Hospital, Yangju 11429, Republic of Korea; gilcadet@gmail.com; 3 Department of Physical and Rehabilitation Medicine, Chung-Ang University Gwangmyeong Hospital, Gwangmyeong 14353, Republic of Korea; grit@cauhs.or.kr; 4 Department of Rehabilitation Medicine, Seoul National University Hospital, Seoul 03080, Republic of Korea; 2mdfeki@gmail.com; 5 Department of Orthopaedic Surgery, Seoul National University Hospital, Seoul 03080, Republic of Korea; chs19@snu.ac.kr; 6 Mellowing Factory Co., Ltd., Seoul 06053, Republic of Korea; joonnyonglee@melab.snu.ac.kr; 7 Department of Rehabilitation Medicine, Seoul National University College of Medicine, Seoul 03080, Republic of Korea; 8 Institute of Aging, Medical Research Center, Seoul National University, Seoul 03080, Republic of Korea

**Keywords:** lumbar lordosis, low back pain, sitting position, chair type, desk tasks, inertial sensors

## Abstract

**Background/Objectives:** Lumbar lordotic curvature (LLC), closely associated with low back pain (LBP) when decreased, is infrequently assessed in clinical settings due to the spatiotemporal limitations of radiographic methods. To overcome these constraints, this study used an inertial measurement system to compare the magnitude and maintenance of LLC across various sitting conditions, categorized into three aspects: verbal instructions, chair type, and desk task types. **Methods:** Twenty-nine healthy participants were instructed to sit for 3 min with two wireless sensors placed on the 12th thoracic vertebra and the 2nd sacral vertebra. The lumbar lordotic angle (LLA) was measured using relative angles for the mediolateral axis and comparisons were made within each sitting category. **Results:** The maintenance of LLA (*LLA_dev_*) was significantly smaller when participants were instructed to sit upright (−3.7 ± 3.9°) compared to that of their habitual sitting posture (−1.2 ± 2.4°) (*p* = 0.001), while the magnitude of LLA (*LLA_avg_*) was significantly larger with an upright sitting posture (*p* = 0.001). *LLA_dev_* was significantly larger when using an office chair (−0.4 ± 1.1°) than when using a stool (−3.2 ± 7.1°) (*p* = 0.033), and *LLA_avg_* was also significantly larger with the office chair (*p* < 0.001). Among the desk tasks, *LLA_avg_* was largest during keyboard tasks (*p* < 0.001), followed by mouse and writing tasks; *LLA_dev_* showed a similar trend without statistical significance (keyboard, −1.2 ± 3.0°; mouse, −1.8 ± 2.2°; writing, −2.9 ± 3.1°) (*p* = 0.067). **Conclusions:** Our findings suggest that strategies including the use of an office chair and preference for computer work may help preserve LLC, whereas in the case of cueing, repetition may be necessary.

## 1. Introduction

The lumbar lordotic curvature (LLC), or the inward curving of the lumbar spine, is one of the most prominent morphological characteristics in the human spinal column and is considered a key structural adaptation to bipedalism [1]. There has been ongoing debate over the relationship between LLC and low back pain (LBP), as LLC has both biomechanical advantages and disadvantages [2,3,4,5]. Preservation of LLC contributes to neutralizing shear loads in the soft tissues surrounding the spine [2], enhancing the capacity to bear gravitational force [3], and preventing the posterior migration of the nucleus pulposus [4]. Conversely, an overly increased LLC may lead to excessive pressure on the posterior ligaments and facet joints [5]. Despite this controversy, recent studies strongly support that a decreased LLC is closely related to LBP [6,7]. The harmful effects of flexed lumbar posture, including increased muscle activity and passive strain on the posterior elements of the spine [8,9,10,11,12], are well documented. However, it is the damage to the posterior annulus of the lumbar disc [13], caused by increased disc pressure and posterior migration of the nucleus pulposus [14,15,16,17], that plays a critical role in pain induction. 

While the causes of LBP are multifactorial [18], prolonged sitting is closely associated with LBP [19,20], with studies showing that awkward postures significantly increase the risk of developing LBP [21]. As a flexed lumbar posture, characterized by decreased LLC, is a common problematic posture while sitting, numerous studies have investigated how LLC is affected by various sitting conditions. Furthermore, studies have assessed how LLC is influenced by specific instructions to maintain good posture [22,23,24,25,26,27,28], such as erect/comfortable/slouch, ideal/habitual, and upright/relaxed. These studies have consistently shown that specific instructions to maintain good posture result in a significantly larger LLC compared to that of habitual sitting postures [23,27,28]. Similarly, numerous studies have compared LLC differences across different types of chairs [26,27,28,29,30,31,32,33,34], including office chairs, standard chairs, stools, or kneeling chairs. Most findings suggest that office chairs, especially those with lumbar support, shows the largest LLC [26,27,28,31], whereas stools tend to show the smallest LLC [31,33]. Only a few studies have examined LLC across various task types [34,35], finding less lumbar flexion during typing compared to using a mouse. Despite the extensive research on the impact of sitting conditions on LLC, these existing studies have not focused on the preservation of LLC over time. While sitting is relatively stable, LLC can change over time if the posture is maintained for an extended period. Considering this, investigating not only the magnitude of LLC but also the degree of change over time may be a critical aspect for developing strategies for spinal health maintenance in everyday sitting.

In clinical settings, evaluating LLC has primarily been conducted using radiographic methods [36,37,38,39], which provide the most accurate, skeleton-based assessment. However, radiographic assessment has inherent limitations, such as spatiotemporal constraints and radiation hazards, which prevent its application to various activities over an extended period. Recent advancements in inertial sensors have offered a more accessible solution for evaluating LLC [25,28,40,41,42,43,44,45,46]. Due to their compact size and wireless capability, inertial measurement systems have gained popularity for assessing spinal curvatures in various clinical scenarios [27,43,44,45] and have been validated against traditional gold-standard measures like radiological and optoelectronic motion capture systems [25,40,41,42,43]. The Attitude and Heading Reference System (AHRS), an advanced inertial sensor system, utilizes complex algorithms to provide more accurate and comprehensive data than conventional inertial sensors [47]. By employing AHRS, this study enables effective real-time monitoring of LLC, presenting a promising method for assessing the preservation of LLC in daily sitting conditions.

In this in vivo study on a healthy population, an AHRS was used to investigate the dynamics of LLC over a duration of 3 min during various sitting conditions, categorized into three groups: verbal instructions, chair type, and desk task types. Our hypothesis was that the preservation of LLC, along with its magnitude, might differ significantly across these conditions. The primary aim of this study was to assess the feasibility of using AHRS for evaluating LLC preservation. Additionally, we sought to determine which postures or tasks within each category are more beneficial for preserving LLC.

## 2. Methods

### 2.1. Design

This study is an analytical cross-sectional study conducted in the Biomechanics laboratory of the Seoul National University Hospital. All participants followed the same test protocols conducted over a single day, which included 10 different sitting conditions. This study is reported following the STROBE (Strengthening the Reporting of Observational Studies in Epidemiology) guidelines [48].

### 2.2. Participants

A total of 29 healthy volunteers, comprising 15 men and 14 women, participated in this study. The mean ages for men and women were 32.2 ± 12.6 years and 39.1 ± 14.1 years, respectively. Anthropomorphic characteristics including the age, weight, height, and body mass index (BMI) of each participant were collected and are summarized in Table 1. Participants recruited for this study had neither experienced LBP nor radicular pain radiating beyond the gluteal fold for 12 weeks leading up to the study, and had no history of back disorders, implying that they were expected to have normal ranges of LLA. Exclusion criteria included skin complications at the sensor attachment site, individuals with psychological disorders such as depression requiring regular medication, pregnant individuals, and those unable to participate in the experiment due to external factors. This study was performed in accordance with the Declaration of Helsinki and was approved by the Institutional Review Board of Seoul National University Hospital (IRB No. 1703-174-842). All participants provided informed consent to participate in the study.

### 2.3. Experimental Set-Up

**Figure 1:**
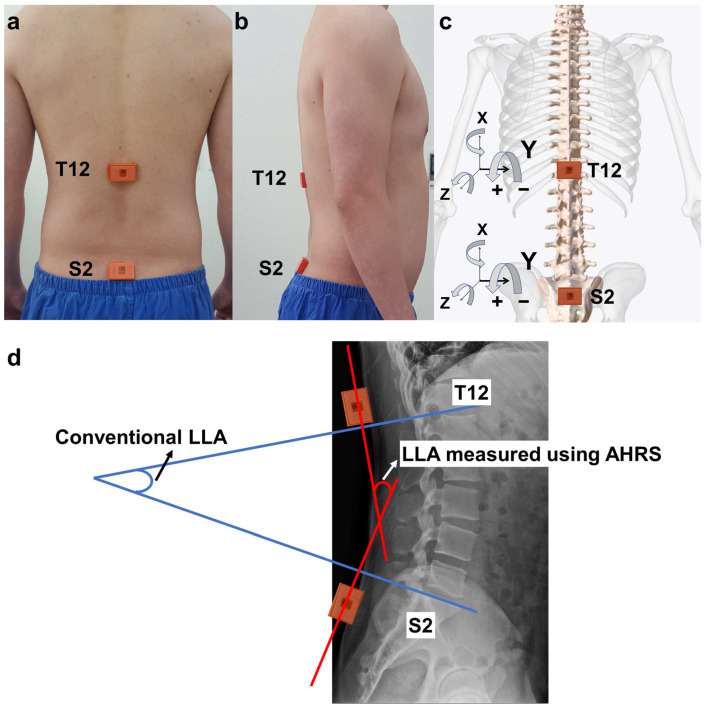
Experimental set-up during the test. (**a**,**b**) IMU sensors were attached to the skin over the 12th thoracic (T12) and 2nd sacral (S2) vertebral points. (**c**) The relative angle between T12 and S2 was calculated using a simple subtraction method, and the *Y*-axis (mediolateral axis) data were used to evaluate the kyphosis–lordosis of lumbar movement between the T12 and S2 IMU sensors. (**d**) LLA is traditionally defined by the Cobb angle measured from the lateral lumbar radiographic images, extending between the inferior endplate of T12 and the superior endplate of S1. The figure illustrates that the measurement derived from this definition is similar to those obtained from IMU sensors.

To evaluate LLC, we used an AHRS based on the wireless inertial measurement unit (IMU, MTw Awinda; Xsens Technologies B.V., Enschede, Netherlands), which uses Strap-Down Integration and the Xsens Kalman Filter to provide more accurate data [47]. Using medical tape, two IMU sensors were affixed on the skin over the spinous process of the 12th thoracic vertebra, palpated along the 12th rib, and over the 2nd sacral vertebra at the midpoint between the posterior superior iliac spines (Figure 1) [49,50]. Prior to data collection, each sensor was calibrated in parallel in the corner of the experiment room to ensure accurate angle measurements. The three output angles were initialized to zero, such that when the two sensors were subtracted after calibration, the angular differences were near zero. Data from the sensors were sampled at a rate of 40 Hz and wirelessly transmitted to a data-processing computer.

### 2.4. Data Processing

The lumbar lordotic angle (LLA) served as a measure for evaluating LLC. Conventionally, the LLA is derived from radiographic images using the Cobb angle, formed between the lines at the inferior endplate of T12 and the superior endplate of S1 (Figure 1d) [50]. When measuring with inertial sensors, the placement can vary slightly among studies; we adhered to the protocols of previously related studies [42,51].

Data processing was conducted in accordance with the methods of our group’s previous biomechanical study [43,46]. The sensor output data provided the 3-dimensional orientations relative to the rotation angles around the X (upward longitudinal axis), Y (mediolateral axis), and Z (anteroposterior axis) axes (Figure 1c). Lumbar posture was determined by subtracting the angles between the thoracic sensor (T12) and the sacral sensor (S2) along each of these axes, and the calculated relative angles along the X, Y, and Z axes correspond to the degrees of axial twist, kyphosis–lordosis, and lateral bending, respectively. Specifically, the *Y*-axis angle, which measures the degree of kyphosis–lordosis, was used as the LLA (Figure 1d). Positive and negative values indicated lordotic and kyphotic alignment of the lumbar spine, respectively. Instead of calculating 3-dimensional Euler angles between the two sensors, a simple subtraction method was utilized to calculate relative angles because the sensors were expected to be moving mainly within the sagittal plane during the test conditions in this study.

### 2.5. Test Protocol

**Figure 2:**
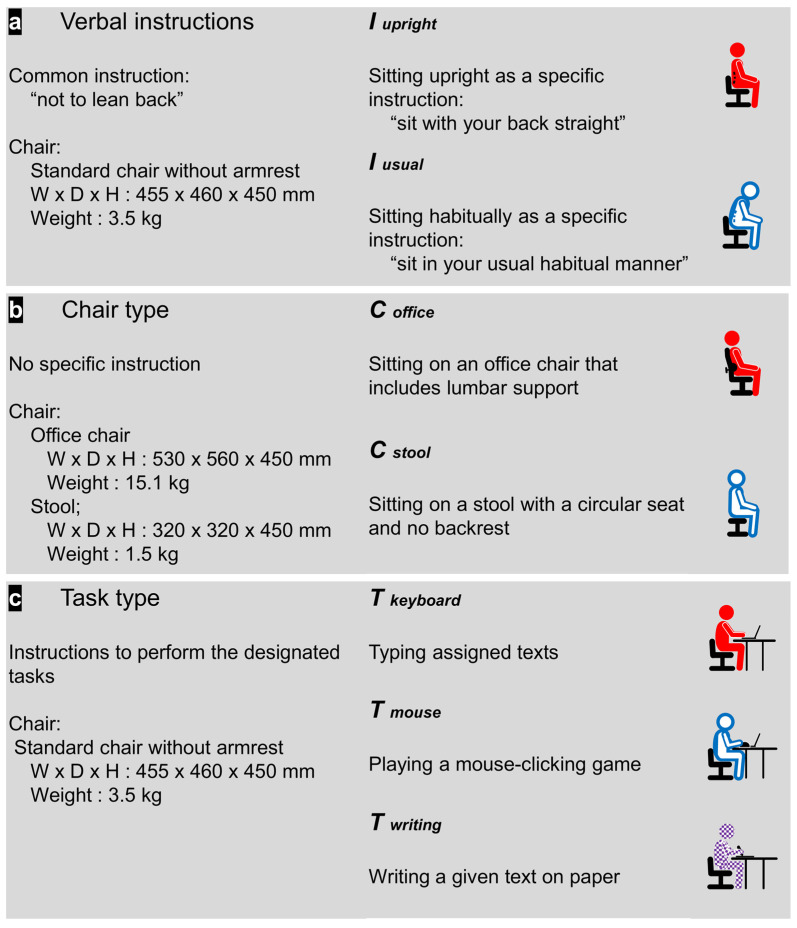
Sitting conditions with the three categories evaluated in the present study. (**a**) Two sitting postures with different instructions, (**b**) two sitting postures on different chairs, and (**c**) three desk tasks. W: width, D: depth, H: seat height.

With the sensors attached to the bony landmarks, participants were asked to undergo seven test conditions, categorized into three aspects: verbal instructions, chair type, and type of desk tasks (Figure 2). We primarily aimed to include postures frequently used in daily life within each category, specifically selecting those where the magnitude of LLA is clearly distinct, to investigate whether maintenance of LLA shows similar trends across these postures.

For the verbal instructions, we chose to compare postures following good posture instructions to those in their usual, habitual state, selecting common directives likely to be encountered in daily life. Specifically, the static sitting postures were performed with the common directive of not leaning back, following two different instructions: to ‘sit with your back straight’ (*I_upright_*) or to ‘sit in your usual habitual manner’ (*I_usual_*). The chair type category included two static sitting postures using different chairs: an office chair (*C_office_*) or a stool (*C_stool_*), both frequently used in daily settings and selected for their potential to show the greatest differences in the magnitude of LLA [31]. Due to limited research on task type, we incorporated a writing task in addition to the tasks utilized from past studies [35]. Desk tasks were performed, including keyboard typing, during which participants typed assigned texts (*T_keyboard_*); computer mouse manipulation, in which participants played a mouse-clicking game (*T_mouse_*); and writing on paper, in which participants transcribed a given text (*T_writing_*). All sitting conditions, except for the chair type category, were performed on a standard chair.

All sitting conditions, except for the chair type category, were performed on a standard chair. The standard chair used was 45 cm in height without armrests. The office chair included lumbar support and was height-adjustable, set to match the height of a standard chair. The stool was 45 cm high with a circular seat and lacked a backrest.

### 2.6. Outcome Variables

**Figure 3:**
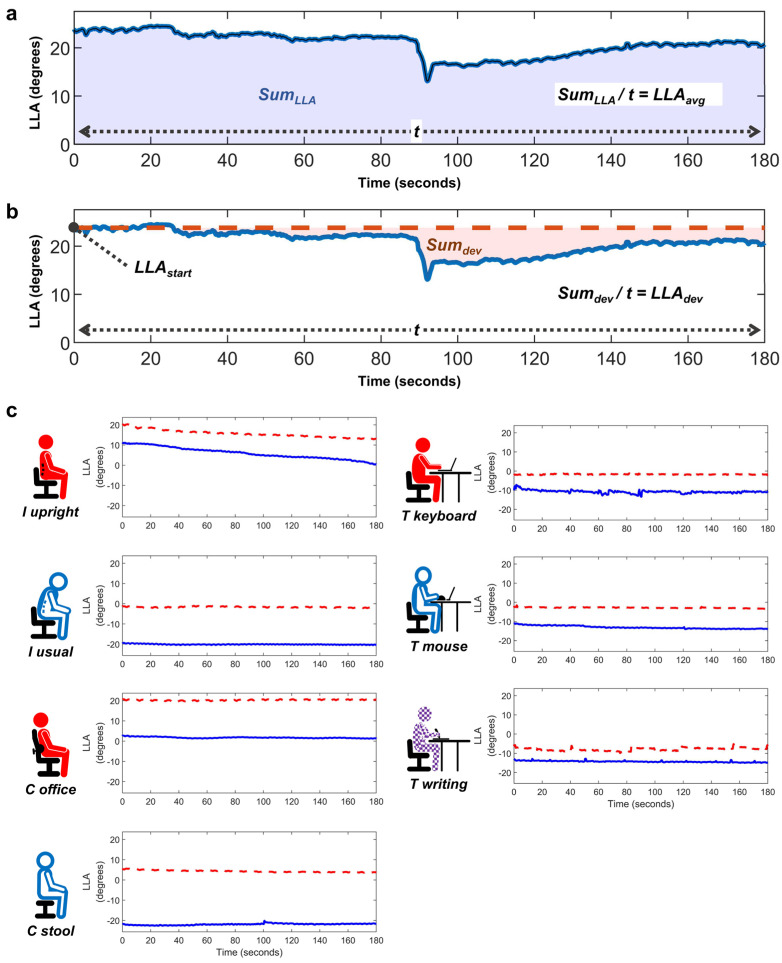
Typical graphs of LLA variation over time. (**a**,**b**) Graphs depicting the LLA changes in a 54-year-old female participant (R007) in a sitting posture on a stool. These graphs illustrate the definition of *LLA_avg_* (**a**) and *LLA_dev_* (**b**). (**c**) Examples of typical LLA variation graphs in different sitting conditions from two participants: a 27-year-old female (R004, red dotted line) and a 49-year-old male (R012, blue solid line).

To assess the magnitude and preservation of LLA, data were tracked for 3 min and displayed as graphs (Figure 3). The primary outcomes included two key variables derived from LLA: *LLA_avg_*, representing the overall magnitude of LLA, and *LLA_dev_*, representing the preservation of LLA. *LLA_avg_* was computed by dividing *Sum_LLA_* by the test duration, where *Sum_LLA_* represents a time-weighted integration of LLA obtained using the trapezoidal rule (Figure 3a). *LLA_dev_* was defined as the deviation from *LLA_start_*, the initial LLA value at the start of the test. It was calculated by integrating the difference between each time point’s LLA value and *LLA_start_* over the total time and then dividing this sum of deviations (*Sum_dev_*) by the test duration. *LLA_dev_* reflects the extent of change in LLA throughout the test duration (Figure 3b). Since most tests showed a decreasing tendency over time, a larger *LLA_dev_* indicates good maintenance of LLC, while a smaller value suggests a greater shift towards a kyphotic position.

### 2.7. Statistical Analysis

The primary analysis involved comparing the primary outcomes (*LLA_avg_* and *LLA_de__v_*) within three sitting categories. Based on the results of the Shapiro–Wilk normality tests, parametric methods were applied for *LLA_avg_*, and non-parametric methods were used for *LLA_de__v_*. Each of the two static sitting postures, with different instructions and those on different chairs, were compared using either paired t-tests or Wilcoxon signed-rank tests. Repeated Measures ANOVA (RM-ANOVA) or Generalized Estimating Equations (GEEs) were used to compare each of the three desk tasks, with post hoc analysis performed using Bonferroni correction.

As a secondary analysis, we aimed to determine if these changes varied by sex, necessitating the use of statistical methods appropriate for repeated measures within two groups. Accordingly, RM-ANOVA or GEEs were used for between-group comparisons and interaction effect analysis for all sitting categories. Within each sex group, comparisons across sitting conditions were made using paired t-tests, Wilcoxon signed rank tests, RM-ANOVA, or Friedman tests, as appropriate. Data are presented as mean ± standard deviation. Statistical analyses were performed using statistical software (SPSS Version 19.0, SPSS Inc., Chicago, IL, USA).

## 3. Results

### 3.1. Comparison within Verbal Instruction Category

**Figure 4:**
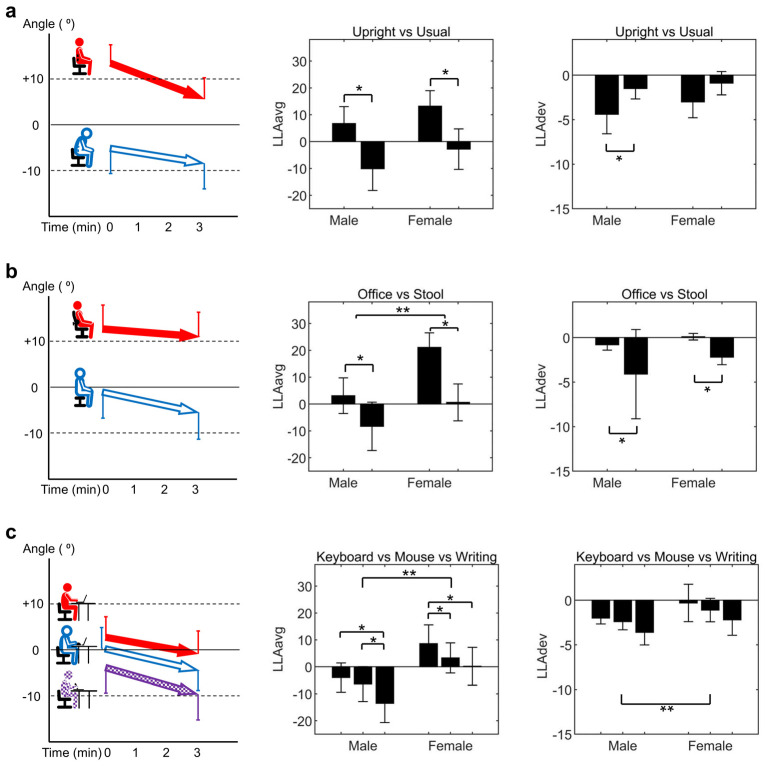
Comparative results within three sitting categories: (**a**) verbal instruction, (**b**) chair type, and (**c**) task type. The first graph in each set depicts the overview of magnitude and temporal changes in LLA, with the start of the arrows denoting *LLA_start_*, and the arrowheads indicating the minimal LLA observed during the test. The second and third bar graphs within each set compare *LLA_avg_* and *LLA_dev_* between sitting conditions within categories and by sex. Single asterisks indicate statistical significance within sex groups across sitting conditions. Double asterisks indicate statistical significance between sex groups within each sitting category. The whiskers represent the 95% confidence intervals and are depicted in a unidirectional manner for clear graphical representation in the first graphs.

Figure 4a illustrates the comparison between upright sitting (*I_upright_*) and habitual sitting (*I_usual_*). The *LLA_avg_* of *I_upright_* (9.9 ± 12.0°) was significantly larger compared to that of *I_usual_* (−6.6 ± 15.4°) (*p* < 0.001, Cohen’s d = 1.6, power = 1.00). The *LLA_dev_* of *I_upright_* (−3.7 ± 3.9°) was significantly smaller compared to that of *I_usual_* (−1.2 ± 2.4°) (*p* = 0.001, r = 0.59, power = 0.85), indicating poorer preservation of LLA in the *I_upright_* compared to *I_usual_*.

### 3.2. Comparison within Chair Type Category

Figure 4b displays the comparison between sitting in an office chair (*C_office_*) and on a stool (*C_stool_*). The *LLA_avg_* of *C_office_* (11.8 ± 14.8°) was significantly larger than that of *C_stool_* (−4.0 ± 16.0°) (*p* < 0.001, Cohen’s d = 1.1, power = 0.99). The *LLA_dev_* of *C_office_* (−0.4 ± 1.1°) was significantly larger than that of *C_stool_* (−3.2 ± 7.1°) (*p* < 0.001, r = 0.44, power = 0.73).

### 3.3. Comparison within Task Type Category

Figure 4c shows the comparison between the three desk tasks. There were significant differences among the three tasks on the desk in *LLA_avg_* (*p* < 0.001, $\eta_{p}^{2}$ = 0.388, power = 1.00). Post hoc analysis revealed significant differences for all three comparisons, with *T_keyboard_* (2.0 ± 13.4°) being larger than *T_mouse_* (−1.7 ± 12.6°) (*p* = 0.009), *T_keyboard_* being larger than *T_writing_* (−6.9 ± 15.3°) (*p* < 0.001), and *T_mouse_* being larger than *T_writing_* (*p* = 0.014). The *LLA_dev_* trends for the three desk tasks were similar to those observed for *LLA_avg_*, but the differences were not statistically significant (*p* = 0.067). Specifically, *LLA_dev_* was −1.2 ± 3.0° for *T_keyboard_*, −1.8 ± 2.2° for *T_mouse_*, and −2.9 ± 3.1° for *T_writing_*.

### 3.4. Sex-Related Differences in LLA across Sitting Conditions

Table 2 presents detailed data on LLA variables, while Table 3 summarizes the results from the comparative analysis conducted using RM-ANOVA or GEE. In the verbal instruction category, no sex-related differences or interaction effects were found for either *LLA_avg_* or *LLA_dev_*. Within each sex group, *I_upright_* showed a significantly larger *LLA_avg_* compared to *I_usual_* in both groups (*p* < 0.001, Cohen’s d = 1.4, power = 1.00 for male, and *p* < 0.001, Cohen’s d = 2.0, power = 1.00 for female). The trend of smaller *LLA_dev_* in *I_upright_* was consistent across sexes but was significant only in the male group (*p* = 0.009, r = 0.78, power = 0.87).

In the chair type category, females exhibited a significantly larger *LLA_avg_* than males (*p* = 0.006, $\eta_{p}^{2}$ = 0.251, power = 0.83), with no interaction effects observed. Meanwhile, no sex-related differences or interaction effects were found in *LLA_dev_*. The *LLA_avg_* of *C_office_* was significantly larger than that of *C_stool_* in both sexes (*p* = 0.013, Cohen’s d = 0.73, power = 0.85 for male and *p* < 0.001 Cohen’s d= 1.72, power = 1.00 for female), and the *LLA_dev_* of *C_office_* was also significantly larger than that of *C_stool_* (*p* = 0.05, r = 0.35, power = 0.35 for male and *p* = 0.001, r = 1.66, power = 1.00 for female).

In the task type category, female participants had a significantly larger *LLA_avg_* and *LLA_dev_* than male participants (*p* = 0.010, $\eta_{p}^{2}$ = 0.221, power = 0.76 for *LLA_avg_* and *p* = 0.032, Wald χ^2^ = 4.6, power = 0.52 for *LLA_dev_*), with no interaction effects observed for either variable. The difference in *LLA_avg_* across the three desk tasks was consistent within each sex group, though statistical significance was only confirmed in some comparisons. 

## 4. Discussion

Utilizing the inertial measurement system, this study aimed to investigate the differences in the magnitude and preservation of LLC within three sitting categories: verbal instructions, chair type, and task type. The analysis revealed that the instructed upright sitting posture resulted in a greater magnitude of LLC compared to that of the usual sitting posture, yet the preservation of LLC was better with the usual sitting posture than that of the upright sitting posture. Regarding the chair type, using an office chair, compared to a stool, contributed to a larger and better maintained LLC. In the task type category, the magnitude of LLC was significantly larger in the order of keyboard use, mouse manipulation, and writing tasks, with a similar pattern noted for the preservation. 

The study’s results show that LLA changes with different instructions, as participants displayed a significantly larger LLA when instructed to sit upright compared to their usual sitting posture. This result is similar to those of previous studies, which showed less flexed positions with the instruction to sit upright [22,26,28]. Particularly, O‘Sullivan et al.’s study, which used a strain gauge-based device, compared habitual and upright sitting [23]. They found that the habitual sitting posture had about a 20% more flexed range of motion compared to upright sitting. It was reasonably expected that a larger LLA would be observed when sitting upright as opposed to habitual sitting. But interestingly, our results suggest that a single instruction to sit upright may not be sufficient to maintain LLA for an extended period, as the decreasing tendency of LLA was significantly larger. While the *LLA_avg_* was significantly different over the 3 min duration used in this study, the difference between the two postures might not have been as distinct if the research had been conducted over a much longer duration. These findings imply that maintaining LLA in daily life may require repetitive feedback rather than a one-time instruction, and they support the potential need for biofeedback devices to aid in preserving LLC [52,53].

Our results, upon comparing the sitting postures involving different chairs, demonstrated that the office chair led to a larger LLA than the stool, a finding consistent with previous research [26,28,32,33]. Particularly, similar outcomes were observed in a radiographic study of 30 healthy males across five sitting positions, which showed the largest LLA when sitting on a chair with back support (36.2 ± 8.4°) and the smallest when sitting on a stool (0.6 ± 3.6°) [31]. A study by Alamin et al., involving 20 asymptomatic subjects, also found significantly smaller LLA when sitting on a stool (16.6 ± 15.6°) compared to when seated on a hard-back chair (28.6 ± 14.3°), similar to our study’s standard chair [33]. One notable aspect of the LLA pattern in the office chair was that it resulted in the largest *LLA_dev_* among all the tests, suggesting that it may be the most advantageous during prolonged sitting.

While several studies have linked prolonged sitting among office workers to LBP [19,21,54], there have been very few that have compared LLA with different types of desk work. Our study revealed that keyboard typing resulted in a larger LLA compared to mouse tasks, a finding consistent with previous research [35]. Additionally, our results showed that both computer tasks, typing and using a mouse, exhibited larger LLA than the writing task. This difference in LLA between tasks can be attributed to the nature of the tasks themselves. In computer tasks, the gaze direction typically involves looking at a monitor, which may encourage a more upright posture, while in writing tasks, the gaze direction is generally directed downwards towards the desk, potentially encouraging more flexion. Another key finding of our study is that there was similar tendency in *LLA_dev_* among the three tasks, with computer work better maintaining LLA compared to writing. This finding is consistent with previous research that suggested postural changes were more prevalent during reading compared to computer work [34].

Numerous previous studies, along with a recent systematic review, have established an association between LLA and sex [55,56,57,58], demonstrating that LLA tends to be larger in females. In our study, a similar tendency was observed across all sitting conditions, with significant differences particularly noted in the chair and task type categories. Notably, some research has indicated that the tendency for a larger LLA in females is posture-dependent, being significant while sitting but not standing [59]. Furthermore, a study focusing on sex-related differences in spine curvature during prolonged sitting on various chairs observed that males exhibited a more flexed lumbar angle than females [35]. However, our research did not find any interaction effects, indicating that being female did not result in disproportionately larger differences across instructions, chair types, or tasks. In terms of preserving LLA, females showed a tendency to better maintain LLA, significantly when performing different type of tasks. This may suggest that the sex-related differences in LLA during sitting are not solely due to skeletal structure differences.

In our study, the preservation of LLA varied across different sitting conditions, with significant differences observed in some categories. This variation in LLA maintenance could primarily be attributed to the inherent characteristics of each sitting condition. However, it may also be influenced by biomechanical factors such as paraspinal muscle activity, which includes considerations of muscle fatigue or the unconscious effort to maintain posture with minimal energy expenditure. Paraspinal muscles, including the erector spinae and multifidus, primarily function as lumbar extensors and play a crucial role in spinal stabilization and motion [60]. Notably, a reduction in the multifidus muscle mass is known to be associated with LBP [61]. O‘Sullivan et al. have demonstrated increased muscle activity during upright sitting compared to slump sitting, which may relate to our findings of insufficient maintenance in upright postures [62,63]. The improved maintenance observed in an office chair in our study could be related to findings from Makhsous et al. indicating that paraspinal activity decreases with the application of lumbar support [27,64]. However, regarding different task types, previous studies have shown lower paraspinal activity during reading compared to computer work, contradicting our hypothesis [34,65,66]. This suggests that the nature of the task itself has a greater influence, indicating that the preservation of LLA results from the complex interplay of various factors. Future research should involve between-subject comparisons to identify biomechanical parameters correlated with LLA maintenance, including paraspinal muscle activity.

Maintaining LLC may be a crucial biomechanical correction to LBP. Several studies have confirmed that modifying sitting posture can directly reduce LBP [67,68,69]. Research, such as the work by Williams et al., highlights that a lordotic posture, when compared to a kyphotic one, can significantly mitigate pain intensity in individuals experiencing LBP [67]. Similarly, studies by Aota et al. and Pillastrini et al. have demonstrated that lumbar supports and ergonomic interventions that help preserve lumbar lordosis significantly reduce the incidence and severity of LBP [68,69]. These studies suggest that strategies to maintain LLC during sitting postures may help prevent LBP. However, they did not directly assess LLA. Therefore, understanding how changes in pain directly relate to variations in LLA, i.e., the relationship between LLC and LBP, has been difficult. Further research utilizing our study methods to directly investigate how specific changes in LLA can prevent LBP is necessary.

Our study has some limitations. First, the short test duration of 3 min is considerably brief compared to that of typical daily sitting activities. Although we incorporated everyday activities into our research for comparison, this short duration makes it challenging to fully generalize our results to actual daily activities. The experimental design, which includes serial conduction of multiple conditions without randomization and the brief resting periods used in our experiment, also make it difficult to confidently apply these findings outside the laboratory setting. These design choices were made to incorporate a variety of test conditions. Future research should consider including fewer test conditions for longer testing durations or multiple trials, sufficient resting periods, and randomization of test condition order. Second, given the cross-sectional nature of our study, it is challenging to establish a direct causal relationship between sitting conditions and LLA changes. Additionally, since the study involved only healthy individuals and did not measure LBP as an outcome, it cannot conclusively prove the preventive effects on LBP. Future research should undertake longitudinal studies or clinical trials to determine whether these strategies can effectively preserve LLC and prevent LBP in real-world applications.

## 5. Conclusions

Consistent patterns and significant differences in magnitude and preservation of lumbar lordotic curvature (LLC) were observed across various everyday sitting conditions among healthy participants using an AHRS. Specifically, within different instructions, the magnitude of LLC was larger in upright sitting, whereas preservation was better in habitual sitting. Comparing chair types, the use of an office chair showed a larger and better maintained LLC compared to using a stool. Within the different task types, the magnitude of LLC was significantly larger in computer tasks than in writing tasks, with a similar trend observed for preservation. These findings suggest that utilizing an office chair and favoring computer work may contribute to the preservation of LLC; however, cueing for upright sitting may require repetition to effectively maintain LLC. Given the association between LLC and low back pain (LBP), these practices could potentially serve as preventive measures against LBP. To further refine our understanding and practical applications of these findings, future research should focus on longitudinal studies or clinical trials, including participants with LBP, that examine the effects of sitting conditions over extended periods to validate the efficacy of interventions aimed at preserving LLC and preventing LBP. Additionally, there is a need for research into the biomechanical factors that influence the preservation of LLC.

## Figures and Tables

**Table 1:** Demographics and characteristics of participants.

	All (*n* = 29)	Male (*n* = 15)	Female (*n* = 14)
Age, years	35.5 ± 13.6	32.2 ± 12.6	39.1 ± 14.1
Body mass, kg	64.0 ± 12.3	72.0 ± 11.9	55.4 ± 4.9
Height, cm	167.1 ± 8.9	173.8 ± 6.0	159.8 ± 4.6
BMI, kg/m^2^	22.8 ± 3.1	23.8 ± 3.3	21.8 ± 2.5

Values are presented as mean ± standard deviation.

**Table 2:** Outcomes of LLA variables by sitting condition and sex.

Sitting Category	Sitting Posture	*LLA_avg_* (°)			*LLA_dev_* (°)		
		All	Male	Female	All	Male	Female
Verbal instruction	*I_upright_*	9.9 ± 12.0	6.7 ± 12.5	13.2 ± 11.0	−3.7 ± 3.9	−4.4 ± 4.3	−3.0 ± 3.4
	*I_usual_*	−6.6 ± 15.4	−10.1 ± 16.0	−2.8 ± 14.4	−1.2 ± 2.4	−1.5 ± 2.3	−0.9 ± 2.5
Chair type	*C_office_*	11.8 ± 14.8	3.1 ± 13.1	21.1 ± 10.3	−0.4 ± 1.1	−0.8 ± 1.2	0.1 ± 0.7
	*C_stool_*	−4.0 ± 16.0	−8.3 ± 17.8	0.6 ± 13.1	−3.2 ± 7.1	−4.1 ± 9.9	−2.2 ± 1.6
Deks tasks	*T_keyboard_*	2.0 ± 13.4	−4.0 ± 10.7	8.6 ± 13.3	−1.2 ± 3.0	−2.0 ± 1.3	−0.3 ± 4.0
	*T_mouse_*	−1.7 ± 12.6	−6.4 ± 12.8	3.3 ± 10.7	−1.8 ± 2.2	−2.4 ± 1.8	−1.1 ± 2.5
	*T_writing_*	−6.9 ± 15.3	−13.5 ± 14.2	0.2 ± 13.4	−2.9 ± 3.1	−3.6 ± 2.8	−2.2 ± 3.3

Values are presented as mean ± standard deviation angles (°).

**Table 3:** Comparisons within sitting categories considering sex-related differences.

Sitting Category	*LLA_avg_* (°)			*LLA_dev_* (°)		
	Item	F	*p*-Value	Item	Wald χ^2^	*p*-Value
Verbal instruction	Instruction	72.159	<0.001 *	Instruction	11.123	0.001 *
	Sex	2.177	0.152	Sex	1.328	0.249
	Instruction × Sex	0.045	0.834	Instruction × Sex	0.221	0.638
Chair type	Chair	38.166	<0.001 *	Chair	4.543	0.033 *
	Sex	9.025	0.006 *	Sex	1.363	0.243
	Chair × Sex	3.080	0.091	Chair × Sex	0.155	0.694
Deks tasks	Task	17.574	<0.001 *	Task	5.402	0.067
	Sex	7.646	0.010 *	Sex	4.611	0.032 *
	Task × Sex	0.959	0.390	Task × Sex	0.133	0.936

* Asterisks indicate statistical significance. (*p*-value < 0.05).

## Data Availability

The data sets for this study are available on request. The raw data supporting the conclusions of this manuscript will be made available by the authors to any qualified researcher. Contact: Ju Chan Kim (ORCID 0000-0003-4015-6096).
